# Validation of an algorithm based on administrative data to detect new onset of atrial fibrillation after cardiac surgery

**DOI:** 10.1186/s12874-020-00953-9

**Published:** 2020-04-05

**Authors:** Jonathan Bourgon Labelle, Paul Farand, Christian Vincelette, Myriam Dumont, Mathilde Le Blanc, Christian M. Rochefort

**Affiliations:** 1 Division of Cardiology, Centre Hospitalier Universitaire de Sherbrooke, Sherbrooke, Quebec, Canada; 2 Faculty of Medicine and Health Sciences, Université de Sherbrooke, Sherbrooke, Quebec, Canada; 3 Research Center, Centre Hospitalier Universitaire de Sherbrooke, Sherbrooke, Quebec, Canada; 4 Research Center, Charles-Lemoyne-Saguenay-Lac-Saint-Jean sur les innovations en santé, Longueuil, Quebec, Canada

**Keywords:** Validation study, Administrative databases, Postoperative atrial fibrillation, Cardiac surgery, Canadian version of the international classification of diseases.

## Abstract

**Introduction:**

Postoperative atrial fibrillation (POAF) is a frequent complication of cardiac surgery associated with important morbidity, mortality, and costs. To assess the effectiveness of preventive interventions, an important prerequisite is to have access to accurate measures of POAF incidence. The aim of this study was to develop and validate such a measure.

**Methods:**

A validation study was conducted at two large Canadian university health centers. First, a random sample of 976 (10.4%) patients who had cardiac surgery at these sites between 2010 and 2016 was generated. Then, a reference standard assessment of their medical records was performed to determine their true POAF status on discharge (positive/negative). The accuracy of various algorithms combining diagnostic and procedure codes from: 1) the current hospitalization, and 2) hospitalizations up to 6 years before the current hospitalization was assessed in comparison with the reference standard. Overall and site-specific estimates of sensitivity, specificity, positive (PPV), and negative (NPV) predictive values were generated, along with their 95%CIs.

**Results:**

Upon manual review, 324 (33.2%) patients were POAF-positive. Our best-performing algorithm combining data from both sites used a look-back window of 6 years to exclude patients previously known for AF. This algorithm achieved 70.4% sensitivity (95%CI: 65.1–75.3), 86.0% specificity (95%CI: 83.1–88.6), 71.5% PPV (95%CI: 66.2–76.4), and 85.4% NPV (95%CI: 82.5–88.0). However, significant site-specific differences in sensitivity and NPV were observed.

**Conclusion:**

An algorithm based on administrative data can identify POAF patients with moderate accuracy. However, site-specific variations in coding practices have significant impact on accuracy.

## Introduction

New onset of atrial fibrillation following cardiac surgery, referred to as postoperative atrial fibrillation (POAF), is a common complication, occurring in approximately 20 to 60% of patients depending on the type of surgical interventions performed [1]. Prior studies have shown that POAF is associated with important morbidity, mortality, and costs [1–5]. Specifically, POAF markedly increases the risk of embolic events (e.g., stroke, thromboembolism), cardiac complications (e.g. heart failure, myocardial infarction, cardiac arrest), as well as renal and respiratory failure [1, 2, 5]. Moreover, POAF doubles the risk of both 30-day and 6-month mortality [1]. In addition, it increases the length of intensive care unit (ICU) and hospital stays by 12 to 24 h and by 2 to 5 days, respectively. Ultimately, longer ICU and hospital stays further increase the cost of a hospitalization [1]. Given these figures, there is a pressing need to better understand the risk factors associated with POAF incidence and to assess the effectiveness of preventive interventions [6]. However, an important prerequisite for meeting these requirements is to have access to an accurate and efficient measure of POAF incidence.

Although manual chart review is the reference standard for identifying adverse events such as POAF [7–11], it is time-consuming, resource-intensive, and costly [12–14]. Other alternatives, such as prevalence surveys or incident reporting systems, similarly lack efficiency and scalability and are well-known for under-reporting issues [12, 15]. For these reasons, there is increasing interest in identifying more efficient and cost-effective methods for monitoring and reporting on adverse events [16].

Among the potential alternatives, administrative data, which are composed of discharge diagnostic and procedure codes, have the advantage of being readily available for large populations of patients, relatively easy to use, and inexpensive [17, 18]. Moreover, several studies have reported that these codes can accurately identify several adverse events, including prevalent cases of AF [2, 7]. However, much less research attention has been given to the development and validation of algorithms for detecting incident cases of AF, such as POAF [19, 20]. Accurately identifying POAF is of particular interest because incident outcomes are more useful than prevalent conditions when conducting surveillance for adverse events or quality improvement initiatives [7]. Moreover, with the growing emphasis on benchmarking and public reporting of adverse events data, detection methods must not only be accurate, they must also allow for valid inter-institutional comparisons [21, 22]. To the best of our knowledge, whether the accuracy of POAF detection algorithms based on administrative data varies across hospitals has never been assessed.

## Methods

### Objectives

The objectives of this multicenter study were to: a) assess the accuracy of an algorithm based on administrative data for identifying patients with incident POAF following cardiac surgery, and; b) determine whether the accuracy of this algorithm varies across sites.

### Study design and population

A validation study was conducted at two large university health centers (UHC) located in the Canadian province of Quebec. A random sample of 986 (10.4%) adult patients aged 18 years and older was selected among 9403 patients who received a cardiac surgery between January 1st, 2010 and December 31st, 2016 at these UHCs (see additional file 1 for more details). Eligible cardiac surgeries are listed in Table 1. These surgeries were selected since they are the most frequent procedures performed, and because they are those of interest for quality / performance assessments in cardiac surgery [23–25]. The eligible surgeries were identified from administrative data using Canadian Classification of Health Interventions (CCI) procedure codes (Table 1) [23]. CCI is a mandatory classification of health-related interventions performed across the care continuum in Canada and is used for physician reimbursement purposes [26].

**Table 1:** Cardiac surgeries of interest

Type of surgery	Intervention codes^a^
Coronary artery bypass graft	1.IJ.76.^^: Bypass, coronary arteries
Valve or annulus procedures	1.HS.80.^^: Repair, tricuspid valve
	1.HS.90.^^: Total excision with reconstruction, tricuspid valve
	1.HT.80.^^: Repair, pulmonary valve
	1.HT.90.^^: Total excision with reconstruction, pulmonary valve
	1.HU.80.^^: Repair, mitral valve
	1.HU.90.^^: Total excision with reconstruction, mitral valve
	1.HV.80.^^: Repair, aortic valve
	1.HV.90.^^: Total excision with reconstruction, aortic valve
	1.HW.^^.^^: Therapeutic interventions on the annulus

^a^Codes are from the Canadian Classification of Health Interventions (CCI) [23].

### Data sources

Depersonalized data were extracted from the MedEcho Discharge Abstract Database (DAD) at the participating sites, which is used for hospital reimbursement purposes and contains mandatory clinical and administrative data on all hospitalizations since April 1, 1976 [27]. For each eligible surgery, we requested DAD data for both the index hospitalization (cardiac surgery) as well as for any prior hospitalizations that occurred at the participating sites in the previous 6 years (see next section for details). For each of these hospitalizations, which were linked using a unique patient identifier (medical record number), DAD provided patient demographics (e.g., age, sex) and clinical characteristics (e.g., principal and secondary diagnoses, surgical procedures performed), and relevant dates (i.e., admission, cardiac surgery, discharge, death). Discharge diagnoses are coded in the Canadian version of the International Classification of Diseases, 10th edition (ICD-10-CA), whereas procedures are coded using the CCI [23]. While both CCI and ICD-10-CA codes are periodically updated, no significant changes were noted during the study period.

### Reference standard development and validation

For each of the randomly selected patients, a reference standard assessment of their medical chart was performed to determine their true POAF status (positive or negative) on hospital discharge. POAF-positive patients were those with: 1) documented evidence of persistent or paroxysmal AF in the period starting immediately after their cardiac surgery and ending on hospital discharge (patients were not followed beyond discharge), 2) no documented evidence of AF in the period starting on hospital admission and ending at the time of the cardiac surgery of interest, and 3) no past medical history of AF as documented in the physicians’ admission notes or any subsequent notes prior to surgery. Patients with transient POAF were considered POAF-negative as were those not meeting the aforementioned criteria. As suggested by Jensen et al. [7], eligible data sources for determining patient true POAF status included, for all patients: 1) discharge summaries, 2) progress notes, 3) electrocardiogram reports, 4) telemetry surveillance reports, and 5) consultants’ notes.

Manual chart review was performed by four blinded medical chart abstractors (MCAs), including three registered nurses with extensive work experience in coronary or surgical intensive care units (JBL, CV, MD) and one postgraduate medical resident (PGY5) in cardiology (ML). Before initiating chart review, MCAs received extensive training and were provided with a standardized data collection tool which was created with the input of a senior cardiologist (PF). The data collection tool was an investigator-developed and password-protected Excel spreadsheet containing: 1) selected patient information to allow for the accurate identification of the sampled charts and hospitalizations (i.e., medical record and hospital visit numbers, hospital admission and discharge dates, patient age and sex); 2) one column for documenting the presence of atrial fibrillation before surgery (yes/no), and; 3) one column for documenting the presence of atrial fibrillation after surgery (yes/no). Each MCA had a distinct list of medical charts to review. Last, to ensure inter-coder agreement, a random sample of 49 (5.0%) charts was rereviewed by a distinct MCA and inter-coder reliability was assessed using Cohen’s Kappa statistics [28]. During chart review, any uncertainties about patients’ true POAF status were discussed among MCAs and resolved through consensus. When necessary, the input of a senior cardiologist (PF) was requested.

### Algorithm development and validation

To develop the POAF detection algorithms, we used discharge diagnostic and procedure codes from the selected cardiac surgery hospitalization as well as from all hospitalizations that occurred at the participating sites in the previous 6 years. A total of six alternative algorithms were developed and tested. While all algorithms included the diagnostic codes listed in Table 2 to flag patients suspected of having POAF, they varied according to: 1) whether the procedure code listed in Table 2 was included or not in the algorithm, and 2) the length of the look-back window used for identifying patients with a history of AF (i.e., 1, 3 or 6 years).

**Table 2:** Discharge diagnostic and procedure codes used in the POAF detection algorithms

Discharge diagnostic codes^a^	I48.0: Paroxysmal atrial fibrillation
	I48.1: Persistent atrial fibrillation
	I48.2: Chronic atrial fibrillation^b^
	I48.9: Atrial fibrillation and atrial flutter, unspecified
	I.48.90: Atrial fibrillation, unspecified
**Procedure codes**	1.HH.59: Maze procedure

^a^Discharge diagnostic codes are from the Canadian version of the International Classification of Diseases, 10th edition (ICD-10-CA), whereas procedures are from the Canadian Classification of Health Interventions (CCI) [23].

^b^Code I48.2 was only used to identify patients with a history of AF and not to suggest the presence of POAF.

Specifically, to be considered POAF-positive, patients needed to have: 1) ICD codes I48.0, I48.1, I48.9 or I48.90 in the discharge abstract of their current hospitalization (in sensitivity analyses, we further examined whether excluding codes I48.9 and I48.90 significantly influenced accuracy), and; 2) no evidence of ICD codes I48.0, I48.1, I48.2, I48.9 or I48.90 is the discharge abstracts of their previous hospitalizations (Table 2).

Based on previous studies [29–31], three different look-back windows were used to identify previous hospitalizations (i.e., 1, 3 and 6 years) and their impact on accuracy was tested in three distinct algorithms. For each of these look-back windows, we further examined (using distinct algorithms) whether having a concomitant maze procedure (i.e., the surgical ablation of chronic AF) at the time of the cardiac surgery of interest influenced accuracy (Table 2). Specifically, patients who concomitantly received a maze procedure were assumed to have a history of chronic AF and were thus coded/recoded as POAF-negatives (even if their discharge diagnostic codes suggested the presence of POAF).

### Patient and hospitalization characteristics

Patient’s age and sex were obtained from discharge abstracts. Comorbidities were measured with the Charlson Comorbidity Index, a weighted index of 17 comorbidities associated with an increased risk of death [32]. Severity of cardiac illness was measured by the Hospital Episode Statistic (HES) score, a prognostic score of both early and one-year mortality based on patient characteristics, comorbidities and prior resource utilization patterns [33]. Last, several characteristics of the current hospitalization were also measured for descriptive purposes from discharge abstract data, including the: 1) type of hospital admission (i.e., elective, semi-urgent, urgent), 2) type of cardiac surgery performed (i.e., coronary artery bypass graft, valvular intervention or both), and; 3) length of hospital stay.

### Statistical analyses

Descriptive statistics were performed to summarize patient and hospitalization characteristics as well as patients’ true POAF status on hospital discharge. The accuracy of each alternative algorithm was assessed in comparison with the reference standard. For each algorithm, estimates of sensitivity, specificity, positive (PPV) and negative (NPV) predictive values were generated, along with their 95% confidence intervals (95%CI) [34–37]. To determine whether any of the algorithms reached statistically significant higher accuracy, McNemar’s test was used to compare their sensitivities and specificities, and Leisenring et al.’s extension of McNemar’s test was employed for comparing their PPVs and NPVs [38]. The threshold of statistical significance for these analyses was fixed at α ≤ 0.05, and Bonferroni’s correction was applied to account for multiple testing. To assess whether accuracy varied across UHCs, in sensitivity analyses, the algorithm with the highest overall sensitivity (i.e., which maximizes case finding) and the one with the highest overall PPV (i.e., which maximizes algorithms’ utility for comparative effectiveness research or benchmarking purposes) were selected. These algorithms were then applied to data from each UHC individually, and their accuracy assessed, as described above. The significance of inter-site differences in accuracy was assessed using McNemar’s test and its extension. All statistical analyses were performed in SAS, version 9.4.

## Results

### Patient characteristics

A total of 986 patients were randomly selected, of which 483 (49.0%) were from UHC A and 503 (51.0%) from UHC B (see Additional file 1). Of these, six patients were excluded since their medical charts were unavailable despite several requests and one because he/she did not receive a surgical procedure of interest (i.e., transcutaneous aortic valve insertion). We further excluded three patients because they died during surgery (and were therefore never at risk for POAF). This resulted in a final sample of 976 patients (479 [49.0%] from UHC A and 497 [51.0%] from UHC B) (see Additional file 1). The characteristics of these patients are listed in Table 3.

**Table 3:** Patient characteristics and outcomes

Patient characteristics	UHC A (***n*** = 479)	UHC B (***n*** = 497)	Total (***n*** = 976)
Age in years – M (SD)	68.4 (9.2)	68.6 (10.7)	68.5 (10.0)
Male sex – n (%)	363 (75.8)	328 (66.0)	691 (70.8)
Charlson score – M (SD)	0.76 (1.21)	1.70 (1.65)	1.24 (1.52)
HES score – M (SD)	4.6 (6.4)	4.6 (7.2)	4.6 (6.8)
Admission type – n (%)			
Elective	256 (53.4)	69 (13.9)	325 (33.3)
Semi-urgent	35 (7.3)	314 (63.2)	349 (35.8)
Urgent	188 (39.3)	114 (22.9)	302 (30.9)
Surgery type – n (%)			
CABG	201 (42.0)	256 (51.5)	457 (46.8)
Valvular	149 (31.1)	143 (28.8)	292 (29.9)
Mixed CABG-valvular	129 (27.0)	98 (19.7)	227 (23.3)
Maze procedure – n (%)	15 (3.1)	16 (3.2)	31 (3.2)
POAF – n (%)			
CABG	59 (29.4)	81 (31.6)	140 (30.6)
Valvular	52 (34.9)	37 (25.9)	89 (30.5)
Mixed CABG-valvular	63 (48.8)	32 (32.7)	95 (41.9)
Total	174 (36.3)	150 (30.2)	324 (33.2)
In-hospital death – n (%)	22 (4.6)	26 (5.23)	48 (4.9)
Length of hospital stay in days - Md (Range)	12 (3–127)	9 (2–217)	11 (2–217)
Number of hospitalizations in the previous 12 months - Md (Range)	0 (0–4)	0 (0–4)	0 (0–4)
Number of ER visits in the previous 12 months - Md (Range)	0 (0–20)	0 (0–4)	0 (0–20)

*Abbreviations: AF*: Atrial fibrillation, *CABG* Coronary artery bypass graft, *ER* Emergency room, *HES* Hospital Episode Statistic score, *M* Means, *Md* Median, *SD* Standard deviation

Several differences in patient characteristics were noted across the two UHCs particularly regarding sex, comorbidity burden, type of hospital admission, type of surgical procedure performed, and length of hospital stay (Table 3). Only 31 (3.2%) patients, equally distributed across the two UHCs, received a maze procedure (Table 3). Upon manual chart review, 324 (33.2%) patients were identified as POAF-positives. The incidence of POAF varied across UHCs, both overall (36.3% vs. 30.2%) and per type of surgical procedures (Table 3). Inter-coder reliability regarding patients’ true POAF status was excellent (κ = 1.0). The median length of hospital stay was 11 days (range: 2–217) and 48 patients (4.6%) died during the postoperative period (Table 3). Compared to UHC B, fewer patients died at UHC A and their median length of hospital stay was longer (Table 3).

### Accuracy of POAF detection algorithms

Table 4 shows the accuracy estimates for each of the six algorithms tested. In summary, using longer look-back windows for excluding patients with a previous history of AF had no impact on sensitivity and marginally increased PPV and specificity (Table 4, Algorithms 1, 3, and 5). Similar patterns were noted when the CCI code for the maze procedure was included in the algorithm (Table 4, Algorithms 2, 4, and 6). However, none of these differences were statistically significant at the 5% threshold. Last, no patient in our sample received ICD code I48.9 “Atrial fibrillation and atrial flutter, unspecified”. Hence, we could not assess whether including this code in the algorithm significantly influenced its accuracy or not.

**Table 4:** Accuracy of different algorithms to detect POAF

	Sensitivity % (95% CI)	Specificity % (95% CI)	PPV % (95% CI)	NPV % (95% CI)
Algorithm 1^a^	70.4 (65.1–75.3)	84.4 (81.3–87.1)	69.1 (63.8–74.0)	85.1 (82.2–87.8)
Algorithm 2^b^	69.4 (64.1–74.4)	85.7 (82.8–88.3)	70.8 (65.4–75.7)	85.0 (82.0–87.6)
Algorithm 3	70.4 (65.1–75.3)	85.4 (82.5–88.1)	70.6 (65.3–75.5)	85.3 (82.4–87.9)
Algorithm 4	69.4 (64.1–74.4)	86.7 (83.8–89.2)	72.1 (66.8–77.0)	85.1 (82.2–87.7)
Algorithm 5	70.4 (65.1–75.3)	86.0 (83.1–88.6)	71.5 (66.2–76.4)	85.4 (82.5–88.0)
Algorithm 6	69.4 (64.1–74.4)	87.3 (84.5–89.7)	73.1 (67.8–77.9)	85.2 (82.3–87.8)

*Abbreviations*: *AF* Atrial fibrillation, *NPV* Negative predictive value, *PPV* Positive predictive value, *95% CI* 95% confidence interval

^a^Algorithm 1, 3, and 5 all included ICD codes I48.0, I48.1 and I48.90 to identify possible cases of POAF. They differed on the look-back window used to exclude patients with a history of AF: 1 year, 3 years, and 6 years for Algorithm 1, 3, and 5, respectively.

^b^Algorithm 2, 4, and 6 all included ICD codes I48.0, I48.1 and I48.90 to identify possible cases of POAF. They differed on the look-back window used to exclude patients with a history of AF: 1 year, 3 years, and 6 years for Algorithm 2, 4, and 6. In these algorithms, all patients who received a maze procedure at the time of their cardiac surgery were considered POAF-negatives.

To assess whether the accuracy of the algorithms varied across UHCs, we first applied the one that achieved the highest overall PPV (Algorithm 6, Table 4) to data from each site individually. In this analysis, both sensitivity and negative predictive value were significantly lower at UHC A, whereas specificity and PPV did not differ between sites (Table 5, Panel A). Similar patterns were observed when applying the algorithm that achieved the highest overall sensitivity (Algorithm 5, Table 4) to data from each site individually (Table 5, Panel B).

**Table 5:** Site-specific accuracy of the best-performing algorithms

Study sites	Sensitivity % (95% CI)	Specificity % (95% CI)	PPV % (95% CI)	NPV % (95% CI)
**Panel A – Site-specific accuracy of the algorithm achieving the highest overall PPV (Algorithm 6)**				
UHC A	61.5 (54.3–68.7)	88.9 (85.3–92.4)	75.9 (68.8–83.0)	80.2 (75.9–84.4)
UHC B	78.7 (72.1–85.2)	85.9 (82.2–89.5)	70.7 (63.8–77.6)	90.3 (87.1–93.5)
**Panel B – Site-specific accuracy of the algorithm achieving highest overall sensitivity (Algorithm 5)**				
UHC A	62.6 (55.5–69.8)	87.9 (84.2–91.5)	74.6 (67.6–81.7)	80.5 (76.2–84.7)
UHC B	79.3 (72.9–85.8)	84.4 (80.6–88.3)	68.8 (61.9–75.7)	90.4 (87.2–93.6)

*Abbreviations*: *CI* Confidence interval, *PPV* Positive predictive value, *NPV* Negative predictive value

## Discussion

POAF is the most frequent complication following cardiac surgery and has major impacts on patient outcomes and healthcare costs. Accurately measuring POAF incidence is important for better understanding its risk factors and for determining the effectiveness of preventive interventions, which are still suboptimal [6]. However, few well-validated and efficient measures of POAF incidence are currently available [7]. The objectives of this study were to develop and validate such a measure using administrative data. In addition, we sought to determine whether the accuracy of this measure was stable across hospitals, which is an important prerequisite for valid benchmarking and comparative effectiveness studies.

We found that patients with POAF can be identified with a reasonable degree of accuracy using an algorithm based on administrative data. Moreover, we noted that the accuracy of this algorithm was not significantly increased by the length of the look-back window (i.e., 1, 3 or 6 years) used for excluding patients with a history of AF from the case definition. This suggests that hospitalizations occurring in the year prior to a cardiac surgery are enough to establish patient baseline status with regards to AF (e.g., presence or absence of chronic AF). In addition, we noted that the accuracy of our algorithms was not significantly enhanced by recoding as POAF-negative all patients who received a concomitant maze procedure at the time of their cardiac surgery and that would have otherwise been identified as POAF-positives based solely on their discharge diagnostic codes. This finding could be attributable to the fact that maze procedures were rarely performed across our study sites.

Indeed, the Canadian Cardiovascular Society Atrial Fibrillation Guidelines recommends that maze procedures should be performed only in patients for which its success is deemed to be high and where additional risks are estimated to be low [39]. Moreover, institutional experience must also be taken into account in the decision to perform this procedure or not [40]. As such, patient characteristics or physician preferences may contribute to explain the infrequent usage of maze procedures at our study sites. By extension, it is reasonable to expect that the impact of including maze procedure codes on algorithm’s accuracy will likely be significant only for very specific subsets of patients or in selected institutions; a hypothesis that should be verified in future studies.

To the best of our knowledge, the algorithms developed and validated in this study are the first specifically targeted at identifying POAF incidence among cardiac surgery patients using administrative data. Moreover, their accuracy compares favorably to that of similar algorithms designed for identifying the incidence of AF among various non-surgical patient populations [19, 20]. Indeed, in a population-based cohort study designed to estimate the incidence and risk factors of AF among whites and African Americans, Alonso et al. reported a PPV of 62% when detecting the incidence of AF using solely discharge diagnostic codes [20]. Likewise, in a case-control study of health maintenance organization enrollees designed to assess compliance rates with antithrombic guidelines and patterns of warfarin use, Glazer et al reported a PPV of 76.8% for a similar detection algorithm [19]. However, none of these studies reported on the sensitivity or specificity of their algorithms. Moreover, both were conducted among non-surgical patients and relied on ICD-9-CM discharge diagnostic codes [7].

Although the general approach to categorizing arrhythmias has not changed from ICD-9 to ICD-10 classifications [7], and even if comparative studies have reported that both classifications generally have similar validity [7, 41], any differences in PPV across studies should nonetheless be interpreted with caution. Indeed, heterogenous patient populations may have different disease prevalence which may influence the PPV of the algorithms tested. Moreover, differences in the characteristics of these algorithms (e.g., number and type of diagnostic codes considered for case definition) and heterogeneity in the reference standards used (e.g., number and type of data sources consulted to determine patient true POAF status) may also contribute to explain differences in PPV (and potentially sensitivity and specificity) across studies [7, 35].

Despite the good overall accuracy of our algorithms, there are important practical implications associated with their performance characteristics. Indeed, our six algorithms achieved moderate sensitivity that ranged from 69.4 to 70.4%. These figures imply false negative fractions of 29.6 to 30.6%, which suggest that the algorithms may miss several cases of POAF. Similarly, among patients with discharge diagnostic codes suggesting the presence of POAF, the observed PPVs imply that between 26.9 and 30.9% of the patients identified as POAF-positive by the algorithms will in fact be disease-free. While such performance metrics may not be appropriate for diagnostic purposes – which is not the intended purpose of the algorithms developed and validated in this study –, they may nonetheless provide useful insights to hospitals interested in following trends and patterns in POAF incidence over time or in monitoring the effectiveness of preventive interventions. The main advantage of these algorithms, over manual chart review, being their high efficiency: they have the ability to scan large amounts of patient records rapidly and at a low cost [42]. However, the results of our sensitivity analyses revealed that hospitals should evaluate their performance characteristics locally prior to using them.

Indeed, we found statistically significant inter-hospital differences in sensitivity and NPV when the algorithms that achieved the highest overall sensitivity (Algorithm 5) or PPV (Algorithm 6) were separately applied to data from UHC A and B. Similar variations in accuracy have been previously observed for algorithms designed to identify various types of adverse events from administrative data [12, 43, 44]. For the most part, these variations have been attributed to differences in reporting and coding practices across institutions [45]. For instance, in some institutions, physicians may more consistently report all cases of adverse events, whereas in others, only unstable cases that required specific treatments and interventions may be documented in the medical chart. Similarly, the degree of thoroughness with which diagnoses are coded may vary between hospitals, and some may code with the objective of maximizing reimbursement instead of reflecting the actual care delivered [46, 47]. All these sources of variations will ultimately influence the performance metrics of an algorithm relying exclusively on administrative data. While several major improvements have been made to coding rules over the years (e.g., higher number of coding fields available, introduction of present-on-admission indicators in certain jurisdictions), our results provide evidence that there is still room for improvement.

To circumvent the limitations of administrative data and increase accuracy, recent studies have used natural language processing (NLP) techniques to supplement adverse event detection algorithms relying on administrative data with additional clinical information only available in narrative format (e.g., progress notes, electrocardiogram reports) [18, 48]. While some of these studies have provided evidence that this approach can significantly increase the accuracy with which prevalent cases of AF are identified [49], clinical narratives and ECG reports were not available to us in an electronic format at the time of this study. Therefore, we could not assess whether including such data increases the accuracy of POAF detection. With the growing availability of clinical narratives in an electronic format, this represents an interesting avenue for further research.

This study has several important strengths, including the use of a large random sample of cardiac surgery patients from two university health centers, the establishment of a reference standard by trained medical chart reviewer who were selected among experienced nursing and medical professionals working in cardiac surgery settings, and the achievement of a high degree of inter-coder agreement which provided a reliable reference standard. Moreover, chart review was performed using a standardized protocol, designed according to the recommendations of prior studies, which suggested that patients’ true POAF status should be determined not solely from 12-lead ECGs, but also from information retrieved across the entire medical chart (e.g., progress notes, reports from consultants) [7]. High inter-coder agreement and the fact that the incidence of POAF observed in our study compares to that reported in previous investigations suggest that few true POAF-positive patients were missed during chart review [4, 28]. Last, examining and reporting on site-specific differences in algorithm’s accuracy represents another strength of this study.

Despite these strengths, our study also has some limitations. First, although it was a multicenter investigation, our two UHCs were sampled in the same jurisdiction (Quebec, Canada). While procedure and discharge diagnostic codes are standard across Canada, they may differ in other jurisdictions, which may reduce the generalizability of our findings. We therefore recommend that our proposed approach be validated in other jurisdictions prior to its use for research or other purposes. Second, although we used several distinct sources of information to document patient true POAF status and define our reference standard, the accuracy of our algorithms is nonetheless contingent on the quality of administrative data, which might be influenced by incomplete or inaccurate documentation of patient POAF status by physicians and/or medical archivists. In addition, it is unknown whether the accuracy/completeness of our reference standard would have been different if we had access to medical records from other hospitals/clinics visited by the selected patients during the 6-year look-back window (and, ultimately, the impact of accessing this data on our algorithm’s accuracy). Further research is required to assess the value-added of accessing this information. Third, time and financial constraints prevented us from having each of the sampled charts reviewed by two independent reviewers, which is the recommended best practice [50, 51]. However, we observed a very high degree of inter-coder agreement on a 5% random sample of the reviewed charts, which suggest that coding errors are probably minimal. Forth, there is a possibility that higher accuracy could have been achieved if we had supplemented our algorithms with NLP-extracted data from clinical narratives [52]. Although we did not have access to such data, it is also important to emphasize that the programmatic resources required to implement NLP techniques are typically not available at most health care institutions, especially in smaller non-academic centers. For these reasons, the approach used in this study represents what is likely achievable at most institutions. Last, although our sensitivity analyses revealed significant differences in algorithms’ sensitivity and NPV across sites, wide confidence intervals suggest that these analyses were underpowered. Therefore, we can’t exclude that significant differences in specificity and PPV would have also been observed if we had access to a larger sample of patients for these analyses.

## Conclusion

POAF can be detected with a reasonable degree of accuracy using an algorithm based on administrative data. Site-specific differences in sensitivity suggest that its performance metrics should be reassessed locally prior to using it as a case-finding tool. However, stable PPVs indicate that the algorithm may prove useful in comparative effectiveness studies or for benchmarking purposes. Future studies should assess whether supplementing this algorithm with NLP-extracted clinical information increases POAF detection accuracy.

## Supplementary information

**Additional file 1.** Flow Diagram of patients included in the study.

## Data Availability

The data supporting the findings of this study were collected at the Centre hospitalier universitaire de Sherbrooke (CHUS) and at the McGill University Health Centre, but restrictions apply to the availability of this data, which were obtained following research ethics and institutional approval at these sites. The data are therefore not publicly available.

## Abbreviations

AF: Atrial fibrillation
CCI: Canadian Classification of Health Interventions
HES: Hospital episode statistic
ICD-10-CA: Canadian version of the International Classification of Diseases, 10th edition
ICU: Intensive care Unit
MCA: Medical chart abstractors
NLP: Natural language processing
NPV: Negative predictive value
POAF: Postoperative atrial fibrillation
PPV: Positive predictive value
UHC: University health center
