# Supplementary material for: Validation of an algorithm based on administrative data to detect new onset of atrial fibrillation after cardiac surgery
Source: BMC Med Res Methodol. 2020 Apr 5;20:75. doi: 10.1186/s12874-020-00953-9 (PMC7132861; doi:10.1186/s12874-020-00953-9)
Supplement: Supplementary file 1 — Additional file 1. Flow Diagram of patients included in the study. [file 12874_2020_953_MOESM1_ESM.docx]

**Additional file 1. Flow Diagram of patients included in the study**

Randomly selected medical charts
(n = 483)

Randomly selected medical charts
(n = 503)

Reviewed medical charts
(n = 479)

Reviewed medical charts
(n = 497)

Charts excluded, with reasons

(n = 4)

- Did not receive a surgical procedure of interest (n=1)
- Died during procedure (n=3)

Medical charts available between January 1, 2010 and December 31, 2016 at UHC A
(n = 3,475)

Medical charts available between January 1, 2010 and December 31, 2016 at UHC B
(n = 5,928)

Final sample

(n = 976)

Charts excluded, with reasons

(n = 6)

- Medical charts inaccessible despite several requests (e.g., physician’s office) (n=6)
